# Integrating geochemical analysis and geospatial techniques to assess groundwater quality and health risks in Wadi Feiran Basin, Southwestern Sinai, Egypt

**DOI:** 10.1038/s41598-025-30127-w

**Published:** 2025-12-17

**Authors:** Ahmed A. Asmoay, Eltaher M. Shams, Abrar Abdel-Salam, Sahar N. E. Tawfik, Rashad Sawires

**Affiliations:** 1 Geological Science Department, Advanced Materials Technology and Mineral Resources Research Institute, National Research Centre, El-Behoos St., Dokki, Giza, 12622 Egypt; 2 Natural Resources and Energy Department, Damanhour University, Beheira, 22511 Egypt; 3 Department of Geology, Faculty of Science, Assiut University, Assiut, 71516, Egypt; 4 Geography and GIS Department, Faculty of Arts, Alexandria University, Alexandria, 21526 Egypt

**Keywords:** Groundwater quality, Surface runoff dynamics, Nemerov’s pollution index, Health risk assessment, Chadha and gibbs diagrams, Wadi Feiran Basin, Southwestern Sinai, Environmental sciences, Natural hazards

## Abstract

This study evaluates groundwater quality in the Wadi Feiran Basin, Southwestern Sinai, by integrating hydrochemical analysis, pollution assessment, and human health risk assessment (HHRA) with morphometric characterization of surface runoff. Morphometric analysis shows that sub-basins vary in runoff potential, reflecting differences in size and topography. Groundwater quality exhibits significant variability, with pH, electrical conductivity, total dissolved solids, and total hardness exceeding World Health Organization (WHO) limits in several samples. Hydrochemical facies analysis indicates that silicate weathering, evaporite dissolution, ion exchange, saline intrusion, and anthropogenic contamination are the dominant processes shaping groundwater chemistry. Pollution assessment using Nemerov’s Pollution Index identifies nitrate and iron as key contaminants, with nitrate exceeding WHO standards in nearly half of the samples. HHRA reveals substantial non-carcinogenic risks, particularly for children, due to elevated nitrate levels, while long-term exposure also suggests potential carcinogenic effects. Overall, 60% of the sampled groundwater is unsuitable for drinking, underscoring the urgent need for monitoring and management strategies to protect public health and ensure sustainable groundwater use in the basin.

## Introduction

**Fig. 1:**
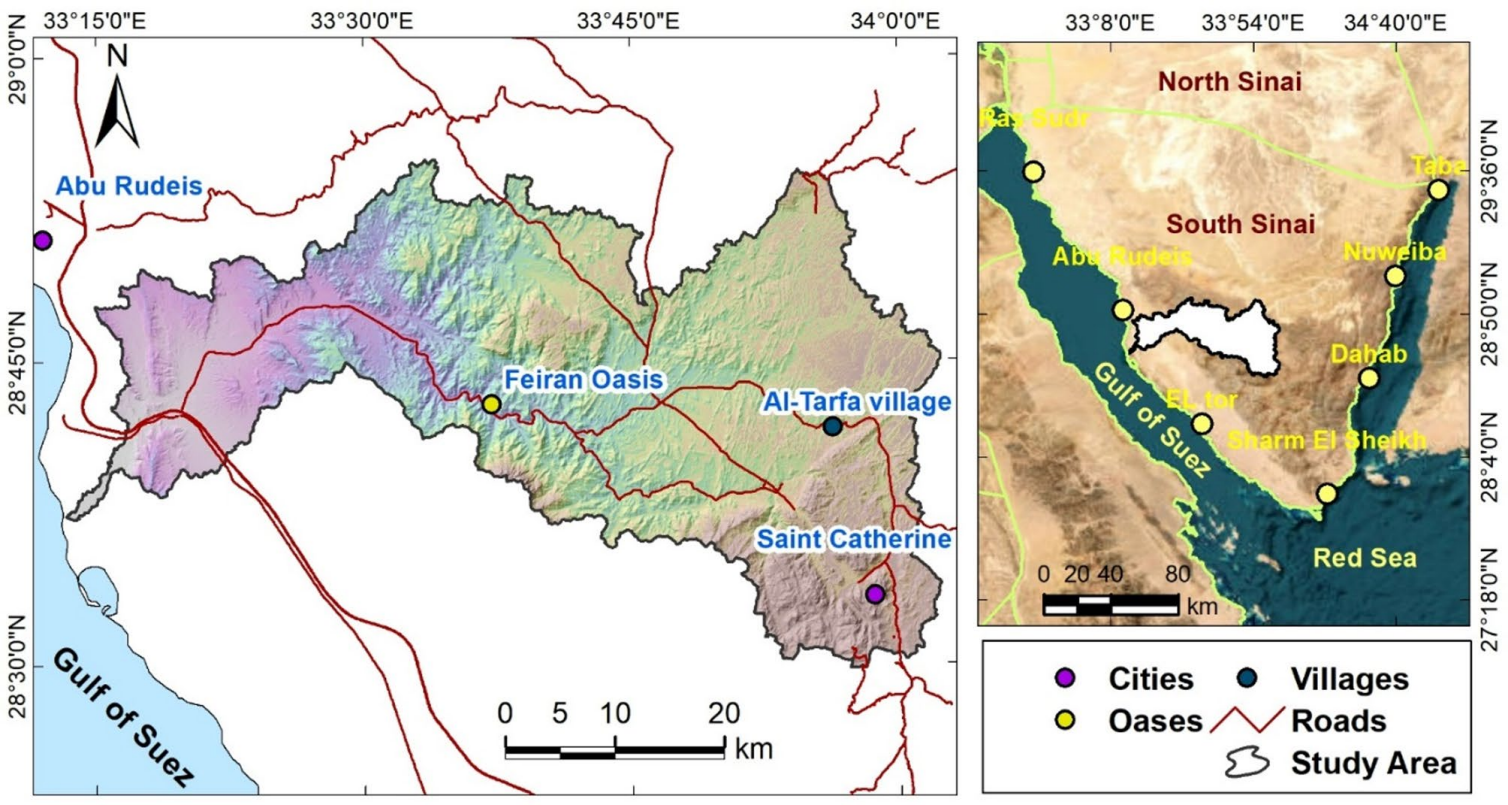
Location map of the Wadi Feiran Basin in southwestern Sinai Peninsula, with main roads and urban centers displayed on a DEM background. The map was created using ArcGIS Desktop v. 10.8. The left panel utilizes base data obtained from the USGS Earth Explorer portal (https://earthexplorer.usgs.gov/), while the right panel features background satellite imagery sourced from Google Earth and integrated into the ArcGIS environment (https://earth.google.com/web/).

In arid and semi-arid regions with little to no surface water, groundwater aquifers are crucial for supporting life and economic activity^1–4^. Located in Southwestern Sinai, about 570 km southeast of Cairo, the Wadi Feiran Basin (Fig. 1) is particularly vulnerable to flash floods, which have historically caused fatalities and substantial property damage^5,6^. In addition to its hydrogeological importance, the area also has profound spiritual and cultural significance, attracting visitors from across the world and contributing to its importance as a destination for spiritual and ecotourism.

Wadi Feiran is the largest and most historically significant valley (or “wadi”) in the southwestern Sinai, valued for both its hydrogeological and cultural importance. Likewise, there is a great deal of potential for groundwater extraction in the Wadi El-Sheikh sub-basin, which is located close to Mount Sinai and Saint Catherine. The varied geological substrates on which both wadis are situated—fractured bedrock, sandstone formations, and unconsolidated alluvial deposits—all play a distinct role in the flow and storage of groundwater^4^. These aquifers are primarily recharged by sporadic flash floods and erratic rainfall that seeps into rock fissures and wadi beds. Despite this potential, the aquifers face growing challenges including over-extraction, contamination, and climate change impacts, underscoring the need for comprehensive assessment^7,8^.

Determining the groundwater’s suitability for different purposes requires a detailed understanding of its chemical makeup in these regions^9,10^. Hydrogeochemical modeling is a powerful tool for clarifying the processes controlling groundwater chemistry, such as evaporation, anthropogenic impacts, and rock–water interactions. Studies carried out in arid areas, such as the southwestern Sinai, have shown that the most common hydrogeochemical processes in wadi aquifers often involve the breakdown of evaporite minerals like gypsum and halite, cation exchange, and recharge water from flash floods^9–11^. These processes contribute to both spatial and temporal variability in water quality.

In Wadi Feiran Basin, groundwater is generally alkaline, with TDS concentrations varying depending on the type of aquifer and recharge circumstances. For instance, alluvial aquifers found in wadi beds typically have lower salinity levels than deeper sandstone aquifers, which can retain dissolved salts for a long time. Recent research has used sophisticated methods like multivariate statistics, inverse geochemical modeling, and isotopic analysis to pinpoint contamination pathways, identify recharge mechanisms, and track the origins of salinity^12–15,85^. Moreover, recent studies have emphasized the necessity of integrated frameworks for assessing water quality that incorporate hydrochemical characterization, pollution indices, and health risk assessments^17,16,18,19^. Since the groundwater in these aquifers is the main supply of drinkable water for the local communities, its integrity is inextricably linked to public health. There are serious risks from contaminants like nitrates, fluoride, and heavy metals that come from both natural geogenic processes and human activity. For example, there have been reports of high fluoride concentrations in some areas of Southwestern Sinai, which may result in fluorosis of the teeth and bones^19,20^. Nitrate contamination is particularly concerned beyond causing methemoglobinemia, nitrates may transform into nitrosamines, which are associated with elevated cancer risks. These issues highlight the need to incorporate HHRA into groundwater studies to account for both carcinogenic and non-carcinogenic exposure pathways.

Despite previous research in Wadi Feiran addressing morphometric analysis, runoff modeling, or hydrochemical characterization individually, no study has yet combined morphometric runoff assessment, Nemerov’s Pollution Index (NPI), and HHRA within a single integrated framework. This gap is significant, as sustainable groundwater management in arid regions requires understanding the interactions between surface processes, groundwater quality, and associated health risks.

This study therefore applies a multidisciplinary approach that integrates morphometric analysis, hydrochemical assessment, pollution indexing, and health risk evaluation in the Wadi Feiran Basin, thereby advancing beyond earlier studies. Specifically, the objectives are to: (1) examine the morphometric characteristics and surface runoff dynamics of the Wadi Feiran Basin, (2) assess groundwater quality using hydrochemical analyses, pollution indices, and multivariate statistical tools, (3) identify the dominant processes controlling groundwater chemistry and contamination, (4) evaluate both carcinogenic and non-carcinogenic health risks associated with groundwater contaminants, and (5) propose strategies for mitigating pollution and protecting groundwater resources for future generations.

By addressing these objectives, the study contributes new insights into hydrogeological and health-related challenges in the Wadi Feiran Basin and provides a foundation for more sustainable groundwater management in arid and semi-arid environments.

##  Historical background

The study area encompasses both the Feiran Oasis and the city of Saint Catherine, each with distinctive geographical, historical, and socio-cultural features. The Wadi Feiran Oasis is located approximately 90 km east of Abu Rudeis city in Southwestern Sinai (Fig. 1). It includes the main Wadi Feiran area and about 50 primary settlements, in addition to around 25 smaller communities. The total population of the oasis and its affiliated settlements is about 8,000 residents, whose water demand is largely dependent on groundwater resources.

While Wadi Feiran is known for its archaeological and religious heritage^21–23^, the present study focuses on socio-economic factors that directly affect groundwater, including population growth, agricultural expansion, and tourism development. These land-use changes have intensified pressures on groundwater extraction and quality, underscoring the importance of sustainable water resource management in the basin.

**Fig. 2:**
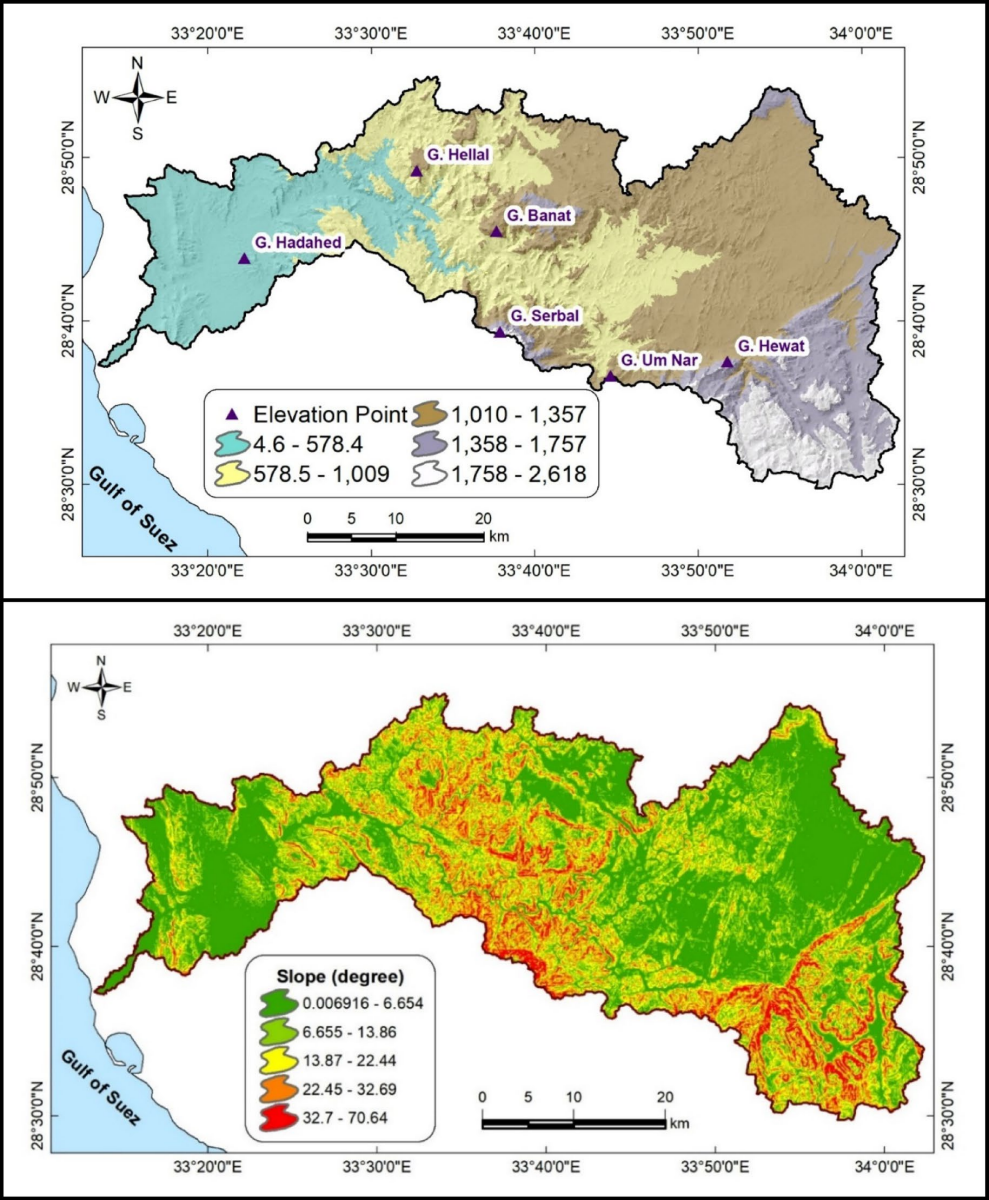
Upper panel presents the DEM in meters, while the lower panel illustrates the slope distribution. The letter“G” in the upper plot stands for “Gebel,” an arabic term that means “mountain.” All maps were produced using ArcGIS Desktop v. 10.8. The upper panel displays a DEM based on elevation data acquired from the United States Geological Survey’s Earth Explorer portal (https://earthexplorer.usgs.gov/).

The city of Saint Catherine (Fig. 1), situated in the heart of Southwestern Sinai atop a high plateau at about 1,600 m above sea level (asl), covers an area of approximately 5,130 km² and is surrounded by Egypt’s tallest peaks—Gebel Saint Catherine, Mount Moses (Gebel Musa), and Gebel Al-Safsafa (Fig. 2). Recognized for its ecological and cultural significance, Saint Catherine was declared a protected natural reserve in 1988. In recent decades, development initiatives, including the ongoing “Great Transfiguration” project, have accelerated tourism and urban growth, further intensifying water demand and groundwater dependence.

## Geological and geomorphological settings

The Wadi Feiran Basin (Figs. 1 and 2) is one of the most prominent drainage systems in Southwestern Sinai. It covers approximately 1,900 km², drains into the Gulf of Suez, and follows an east–west orientation^6,24^. As the largest drainage basin in the Gulf of Suez drainage network, Wadi Feiran plays a crucial role in regional hydrology. The main course, together with its tributaries, drains rugged mountains including Gebel Saint Catherine, Gebel Musa, and Gebel Serbal, which reach elevations up to 2,640 m asl (Fig. 2). The basin traverses geological units ranging from Precambrian to Quaternary^25,26^.

**Fig. 3:**
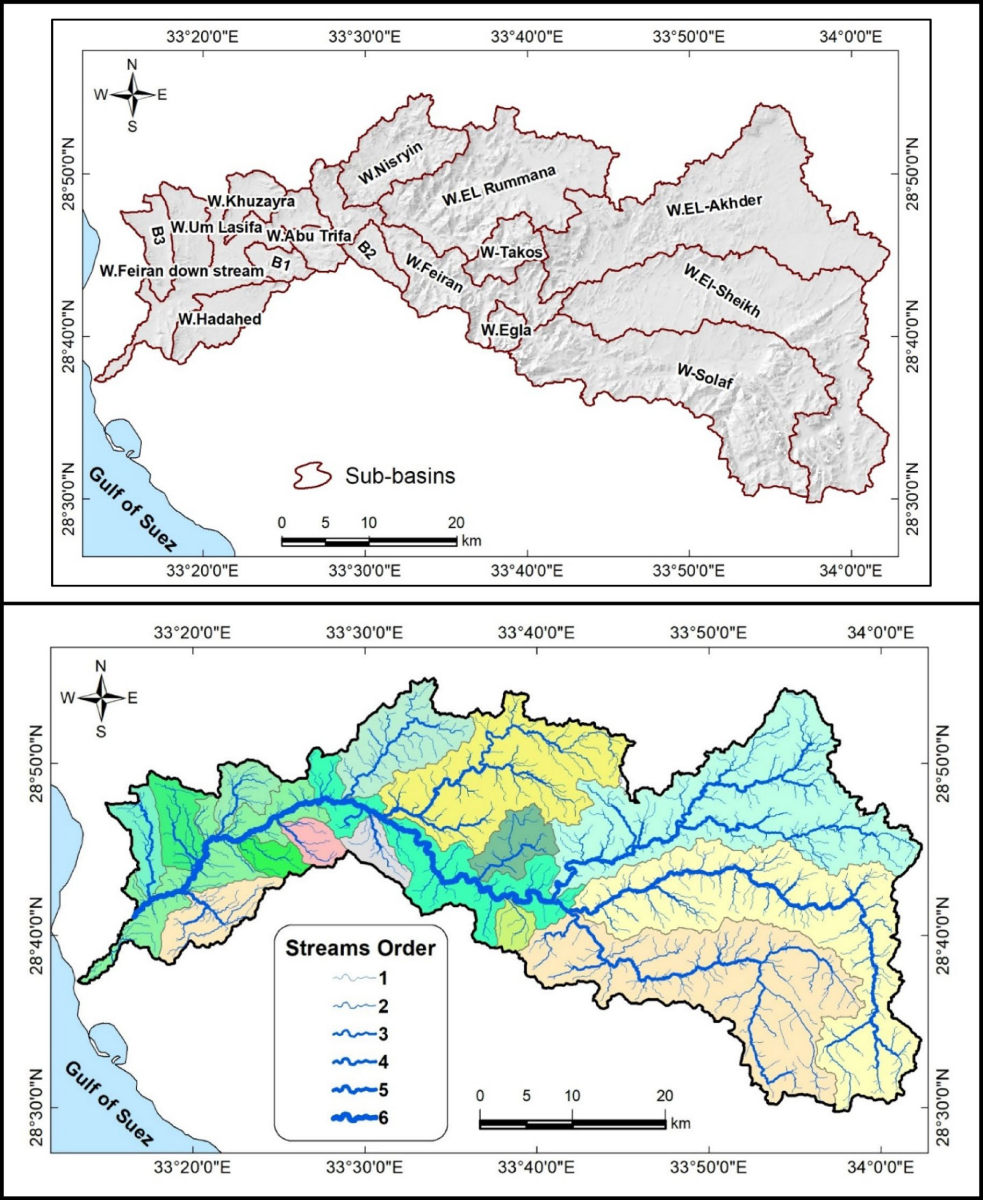

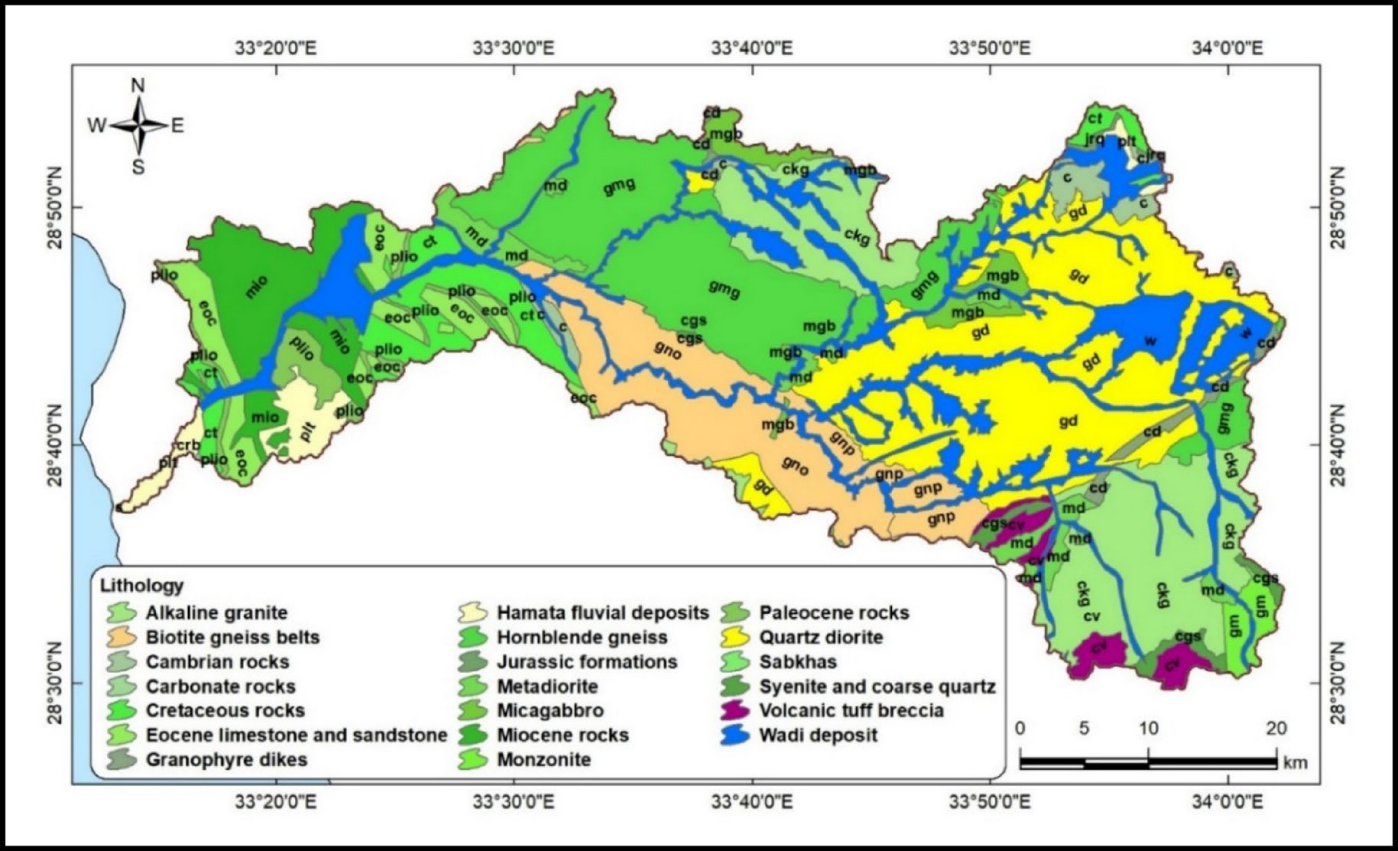
Geospatial map of the Wadi Feiran Basin, showing sub-basin divisions in the upper plot, stream order in the middle plot, and detailed geological map of the study area in the lower plot. All maps were produced using ArcGIS Desktop v. 10.8. The upper panel displays data acquired from the USGS Earth Explorer portal (https://earthexplorer.usgs.gov/). The lower panel features a geological map obtained from the Egyptian General Survey Authority [27].

Geomorphologically, Wadi Feiran can be divided into two main zones: an upper mountainous zone and a lower relief zone (Figs. 1 and 2). The upper zone is dominated by high-relief granitic terrain, including Gebel Musa, Gebel Serbal, and Gebel Banat, composed largely of granites, gneisses, migmatites, and granitoids. The lower zone is characterized by gentler relief and broader tributaries, with exposures of Phanerozoic sedimentary rocks near the basin mouth (Fig. 3)^27^.

Climatically, the region experiences hot summers and mild winters, with observed minimum and maximum temperatures around 13 °C and 34 °C (Catharine Weather Station). Annual precipitation is extremely low (1.6–21.5 mm), with occasional extreme events reaching up to 76.2 mm^28^. Recent studies show that climate change trends in Sinai include rising mean temperatures and increasing rainfall irregularity, contributing to more frequent and intense flash floods^29^,^32^,^31^,^30^ . These changes can reduce effective groundwater recharge by shortening infiltration windows and amplifying surface runoff, thereby intensifying vulnerability of aquifers in arid basins like Wadi Feiran.

Hydrologically, the basin serves as the primary drainage route in Southwestern Sinai. It collects runoff from tributaries such as Wadi El-Akhder, Wadi El-Sheikh, and Wadi Solaf, which converge at the Feiran Oasis. While these floods represent a potential recharge source, they also pose recurring hazards, damaging infrastructure and settlements along the Feiran–Saint Catherine Road^5,6,26^.

## Hydrogeological setting

The Wadi Feiran Basin is underlain mainly by fractured basement rocks, which form the dominant aquifer system (Fig. 3). Groundwater is the sole water supply for the basin’s population, making its assessment critical for sustainable use. Effective exploration and utilization require understanding the interplay of geology, geomorphology, structures, and climate, all of which govern aquifer recharge and storage^2,4–6,8,11,12,20,33–35^.

Several studies have investigated the hydrology and hydrogeology of the basin^5,25,36–39^, Based on stratigraphy (Fig. 3), groundwater occurs in three main aquifers:

- Quaternary Alluvial aquifer: the principal reservoir, composed of sandy gravels and arkosic sandstones. Water table depths range 8–44 m, but recent records show declining levels due to variable recharge and extraction. Recharge occurs mainly via episodic floods and rainfall.
- Pre-Cenomanian Sandstone aquifer: confined in the low-lying western basin, playing a secondary role in storage and movement.
- Pre-Cambrian Fractured Basement aquifer: located in the elevated upstream areas. Permeability is highly dependent on fracturing and faulting, with depths of 4–10 m. Water levels here also show declining trends, reflecting sustainability challenges.

The aquifer descriptions have been streamlined to highlight groundwater sustainability challenges, including declining water levels and recharge limitations. Recent studies on fractured rock aquifers and groundwater sustainability frameworks. They have also been incorporated to provide an updated hydrogeological context that complements the foundational knowledge of the basin^4,6,11,12,20,25,35^.

## Materials and methods

### Spatial data sources and morphometric analysis

To achieve the objectives of this study, a range of spatial datasets were compiled from reliable sources. Topographic maps of the Wadi Feiran Basin, at a scale of 1:50,000, were obtained from the Egyptian General Survey Authority to provide detailed terrain information (Fig. 3). A Digital Elevation Model (DEM) with a spatial resolution of 30 m was downloaded from the United States Geological Survey (USGS) Earth Explorer platform and served as the primary dataset for elevation and terrain analyses. While the 30 m DEM provides adequate coverage for regional-scale geomorphological studies, its limitations include reduced accuracy for fine-scale terrain features, potential errors due to mountain shadowing, and lower precision compared with LiDAR or high-resolution ground surveys. Nonetheless, the DEM selected from USGS is widely used in similar studies and was considered suitable for the rugged mountainous terrain of the basin and the scope of this investigation.

Additional datasets were incorporated to support geomorphological and hydrological characterization. Sentinel-2 satellite imagery (10 m resolution, February 2025) was obtained from the USGS Earth Explorer for land-use and land-cover mapping. The imagery was processed using a supervised classification approach, and classification accuracy was validated through field verification (Fig. 3). Together, these sources provided a comprehensive spatial database for the morphometric analysis of the Wadi Feiran sub-basins.

To assess the hydrological behavior and geomorphological characteristics of the catchment, a suite of morphometric parameters was calculated. These included drainage network, shape, and relief indices, which are summarized with their equations and references in Table 1. Among the shape parameters, the elongation ratio (Re), circularity ratio (Rc), and form factor (Ff) are diagnostic indicators of basin geometry and drainage efficiency. The Re measures how closely a basin’s shape approaches a circle, with lower values indicating elongated basins that typically produce delayed peak flows^40^. The Rc expresses the degree to which a basin resembles a perfect circle, with higher values reflecting compact basins prone to rapid runoff concentration and higher peak discharge^41^. The Ff relates basin area to the square of its length, indicating flood susceptibility: lower values represent elongated basins less prone to flash floods, whereas higher values suggest more circular basins with greater flood risk potential^42^.

**Table 1:** Estimated morphometric parameters and their equations. The parameters are grouped into drainage network, shape, and relief indices, providing a comprehensive framework to evaluate basin geometry, drainage efficiency, recharge potential, and hydrological response.

Category	Parameter	Formula	Reference
Drainage network	Stream order (U)	Hierarchical rank	61
	Stream number (Nu)	Count of streams per order	61
	Stream length (Lu)	Σ length of streams per order	^60^
	Mean stream length (Lsm)	Lsm = Lu/Nu	^60^
	Bifurcation ratio (Rb)	Rb = Nu/Nu + 1	^60^
	Drainage density (Dd)	Dd = ΣLu/A	^60^
	Stream frequency (Fs)	Fs = ΣNu/A	^60^
	Texture ratio (T)	T = ΣNu/P	^60^
Shape	Basin area (A)	Planimetric area	^60^
	Basin perimeter (P)	Length of basin boundary	^60^
	Form factor (Ff)	Ff = A/Lb²	^60^
	Elongation ratio (Re)	Re = (2/Lb) × √(A/π)	^62^
	Circularity ratio (Rc)	Rc = (4πA)/P²	^65^
	Compactness coefficient (Cc)	Cc = 0.2821 × P/√A	87
Relief	Basin relief (H)	H = Hmax – Hmin	^62^
	Relief ratio (Rr)	Rr = H/Lb	^62^
	Ruggedness number (Rn)	Rn = H × Dd	61
	Gradient ratio (Rg)	Rg = H/Lb	^62^
	Relative relief (Rhp)	Rhp = (H × 100)/P	^66^

In addition to these shape indices, other parameters such as drainage density (Dd), bifurcation ratio (Rb), and relief ratio (Rr) were calculated to capture the structural and hydrological controls on basin behavior. Collectively, these indices provide a robust framework for evaluating the relationship between basin morphology, drainage efficiency, recharge potential, and hydrological response.

### Geochemical analysis of groundwater samples

**Fig. 4:**
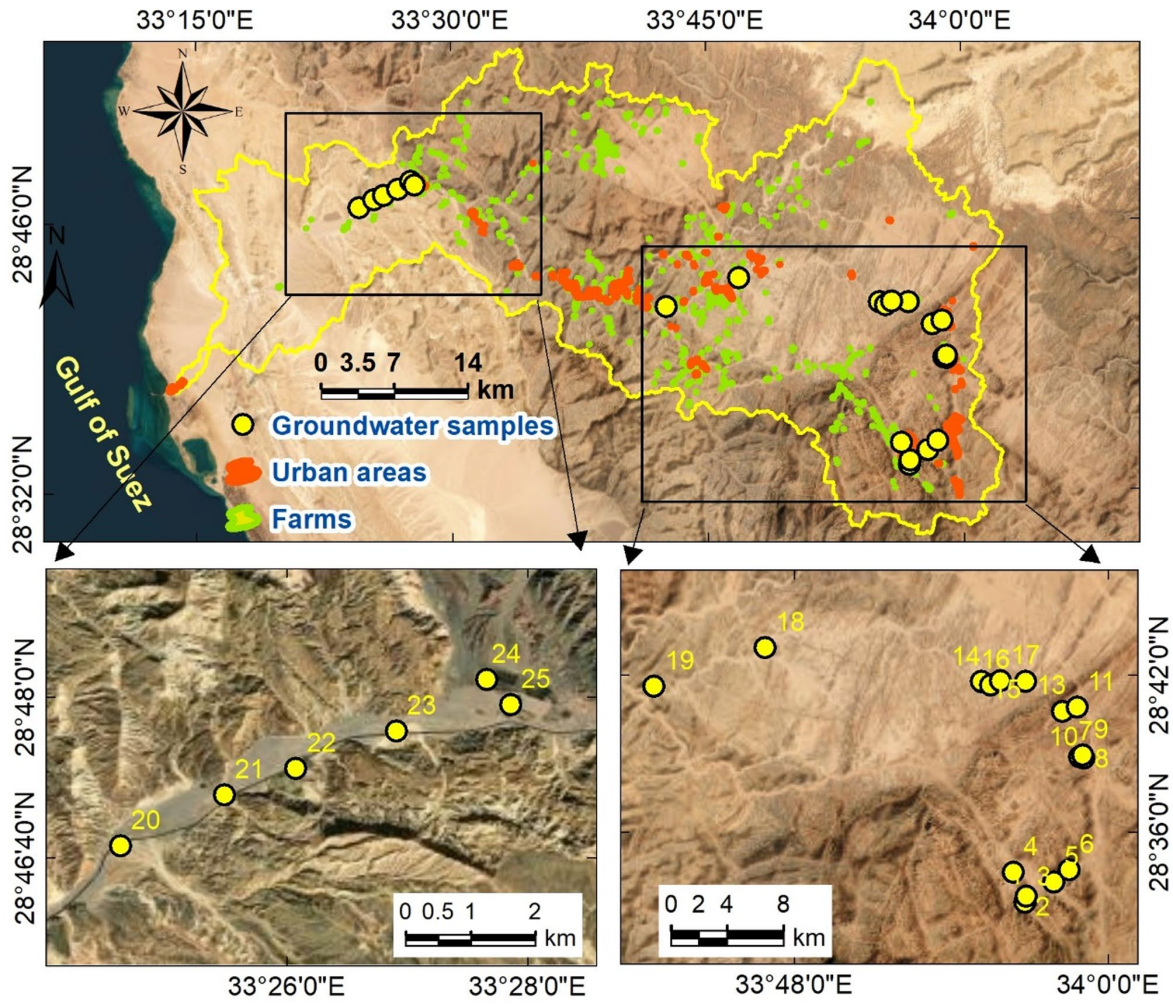
Map showing the locations of collected groundwater samples, primarily situated along the downstream section of Wadi Ferian (west) and Wadi El-Sheikh (east). Maps were generated using ArcGIS Desktop v. 10.8, with background satellite imagery sourced from Google Earth and integrated into the ArcGIS environment (https://earth.google.com/web/).

Twenty-five groundwater samples were taken from wells in the Wadi El-Sheikh and Wadi Feiran sub-basins in the fall of 2024 (Fig. 4). The sites were deliberately chosen to capture the region’s hydrogeological diversity, settlement patterns, and land-use practices. Samples from the Feiran Oasis represent domestic consumption and intensive agricultural activities within nearly 50 major settlements and more than 25 smaller communities, while those from the Saint Catherine area reflect groundwater use associated with rapid urban expansion and tourism development. To guarantee accuracy and comparability, field protocols adhered to APHA guidelines^43^. While major ions (Ca²⁺, Mg²⁺, Na⁺, K⁺, HCO₃⁻, Cl⁻, SO₄²⁻, NO₃⁻, and F⁻) and specific heavy metals were measured in the laboratory, basic parameters (pH, EC, and TDS) were measured on-site right away (Table 2). Ionic balance calculations confirmed that the analytical precision was within ± 5%, indicating the reliability of the data^4,35,85,86^.

**Table 2:** Developed procedure for evaluating significant ions in the groundwater of the study area

Parameter	Method/instrumentation	Reagent
pH	pH-meter hanna USA H-198,130	Potassium chloride (KCl)
EC (µS/cm)		
TDS (ppm)		
TH as CaCO_3_(ppm)	Titrimetric	Hydrochloric Acid (HCl) and standard EDTA solution
Ca^2+^ (ppm)	Titrimetric with EDTA	EDTA, sodium hydroxide (NaOH) and murexide
Mg^2+^ (ppm)	TH-Ca	Calculation
Na^+^ (ppm)	Flame photometer (elico) (systronics, 128)	Sodium chloride (NaCl), KCl and Calcium carbonate (CaCO_3_)
K^+^ (ppm)		
HCO_3_^−^ (ppm)	Titrimetric	Hydrosulfuric acid (H_2_SO_4_), methyl orange
Cl^−^ (ppm)		Silver nitrate, potassium chromate
SO_4_^2−^ & NO_3_^−^ (ppm)	UV–Visible spectrophotometer (Spectronic 21, BAUSCH and LOMB)	Following APHA (2017) standard protocol
F^−^ (ppm)	Ion selective electrode, (Orion analyzer)	TISSB- III, F stock solution
Heavy metals (ppm)	Atomic absorption spectrometry (AAS, perkinElmer 400)	Following APHA (2017) standard protocol

Several methods were employed to assess water facies and geochemical controls: (i) Hydrochemical facies are categorized using Piper and Durov diagrams (AqQA software). (ii) Mineral equilibrium states are evaluated using saturation indices (Visual MINTEQ 3.1). (iii) Multivariate statistics to determine governing processes and sources of pollution (PCA, cluster analysis in GraphPad Prism). This integrated approach is justified by the fact that a robust interpretation of water–rock interaction, contamination pathways, and anthropogenic influences can be obtained by combining graphical, statistical, and geochemical indices.

#### Nemerov’s pollution index (NPI)

By combining the dominant and secondary pollutants, NPI was used to evaluate the overall state of the water quality^44–46^. This approach was selected because it is more representative than single-parameter indices, highlighting the most important contaminant while taking cumulative effects into account. In various hydrogeological contexts, such as China and India, NPI has been extensively utilized for groundwater quality evaluations^45,46^. Its application here offers a clear analogy to recent research conducted in arid areas under comparable stressors. Equations (1–3) were used:

1 $$\:Pi=\frac{\text{Ci}}{\text{Si}}$$

where Si is the corresponding WHO standard limit, Ci is the measured concentration, and Pi is the pollution index for each parameter.

2 $$\:P1=\frac{1}{n}\sum\:_{i=1}^{n}Pi$$

where n is the number of variables taken into consideration, and P1 is the average of all Pi values.

3 $$\:NPI=\sqrt{\frac{{\left(P1\right)}^{2}+{Pi}_{max}^{2}}{2}}$$

where the maximum Pi value is called Pimax. Non-polluted (< 1), slightly (1–2), lightly (2–3), moderately (3–5), and severely polluted (> 5) are the classifications for NPI^45,46^.

#### Human health risk assessment

The US EPA framework was used to assess the health effects of groundwater contamination^47,48^. This approach was chosen due to its ability to distinguish between non-carcinogenic substances by combining the pathways of oral ingestion and dermal exposure (Eqs. 4–7). Nitrate was the focus because of its known health effects and high levels in some areas of the basin^4,49–52^. Hazard Quotient (HQ), Hazard Index (HI), and Chronic Daily Intake (CDI) were among the parameters (Table 3). According to US EPA^47,48^, a benchmark HQ or HI > 1.0 was deemed hazardous to human health.

4 $$HI = HQ~Oral~~ + HQ~Dermal$$

**Table 3:** US EPA standards for the key coefficients in groundwater contamination assessment.

Parameter	Significations	Infants	Children	Adults
R*f*D _Oral_ (mg.kg^− 1^ d^− 1^) for NO_3_	Reference dose of a particular non-carcinogenic substance in water	1.6		
R*f*D _Dermal_ (mg.kg^− 1^ d^− 1^) for NO_3_	Cancer slope factor	0.8		
IR (L.d^− 1^)	Rate of water consumption	0.3	1.2	2
EF (d.a^− 1^)	Frequency of exposure	365		
ED (a)	Duration of exposure	1	12	30
BW (kg)	Weight of residents	7	20	65
AT for non-carcinogenic (d)	Life expectancy of residents	365	4380	10,950
SA (cm^2^)	Skin contact surface area	2.8 × 10^3^	6.6 × 10^3^	1.6 × 10^4^
K_P_ (cm.h^− 1^)	Skin permeability coefficient	0.001		
EV	Frequency of bathing	1.00		
ET (h.d^− 1^)	Bath duration	0.40		
CF (L.cm^− 2^)	Volume conversion factor	0.001		

For identifying vulnerable populations (such as children) and setting priorities for management measures in water-stressed areas, this method offers a quantitative connection between hydrochemistry and public health.

5 $$\:HQ=\frac{\text{CDI}}{\text{RfD}}$$

6 $$CDI~Oral = \frac{{C~.~IR~.~EF~.~ED}}{{BW~.~AT}}$$

7 $$CDI~_{{Dermal}} = \frac{{C~.~SA~.~K_{{P~}} .~EV~.~ET~.~EF~.~ED~.~CF}}{{BW~.~AT}}$$

### Composite risk map generation

To provide an integrated framework that combines morphometric characteristics, surface runoff dynamics, geochemical conditions, and HHRA, the Analytic Hierarchy Process (AHP) was applied. AHP is a widely used multi-criteria decision analysis tool that enables the weighting and integration of diverse spatial factors into a composite risk model^53–55^. This approach allowed us to generate a spatially explicit map highlighting areas most vulnerable to groundwater health risks.

Four thematic layers were selected for integration: (i) distance from watercourses, representing recharge sources and pollutant transport pathways; (ii) distance from urban areas, reflecting anthropogenic pressures and wastewater inputs; (iii) population density, indicating the degree of exposure and community vulnerability; and (iv) nitrate concentration, serving as the key geochemical indicator of health risk. Each layer was standardized to the same coordinate system and resolution and reclassified into comparable categories.

Pairwise comparisons among criteria were performed using Saaty’s 1–9 scale^53^ to construct the comparison matrix. Relative weights were then calculated, and the Consistency Ratio (CR) was applied to ensure that judgments were acceptable (CR < 0.10). The principal eigenvalue was 4.02, very close to the total number of criteria (4.00), confirming matrix stability. The CR was only 0.8%, well below the 10% threshold, indicating a high degree of consistency. The eigenvector solution converged within three iterations with a very small delta (Δ = 6.0 × 10⁻⁸), demonstrating rapid convergence and numerical accuracy. Sensitivity analysis was conducted by varying weights within ± 5% to evaluate the robustness of the model, following similar approaches in groundwater contamination studies^56,57^.

Finally, the weighted overlay technique was applied in a GIS environment to produce a classified composite risk map (very low, low, moderate, and high risk). The use of AHP in hydrogeological studies has been shown to effectively integrate geospatial and geochemical information for environmental risk mapping^3,58,59^, and its application here provided a systematic way to combine natural and anthropogenic factors influencing groundwater quality in the Wadi Feiran Basin.

## Results and discussions

### Morphometric characteristics of the Wadi Feiran sub-basins

The Wadi Feiran Basin comprises 16 sub-basins, each varying in size and geomorphological characteristics (Fig. 3). These sub-basins originate from the mountainous regions of southern Sinai, particularly the Saint Catherine Mountain range. Among them, Wadi El-Sheikh, Wadi El-Akhder, and Wadi Solaf are the largest, with catchment areas of approximately 340, 330, and 310 km², respectively (Table 1). Morphometric analysis was conducted to evaluate drainage network properties, basin geometry, and relief characteristics, as these factors strongly influence hydrological behavior, groundwater recharge, and flood potential^60–62^.

The drainage network parameters (Table 4) revealed clear variability among the sub-basins. Larger catchments, such as El-Sheikh, El-Akhder, and El-Rummana, exhibited the highest stream numbers and lengths, consistent with their extensive drainage networks and elevated erosion rates. In contrast, smaller basins including Egla, Khuzayra, and Um Lasifa contained fewer and shorter streams, reflecting more limited spatial extent and simpler hydrological settings. Drainage density values ranged from 0.40 in Feiran, indicative of highly permeable lithologies and/or arid conditions, to 1.20 in Hadahed, where steeper slopes and less permeable formations promote rapid channel development. Stream frequency followed similar trends, with higher values in densely dissected basins (e.g., El-Sheikh, Hadahed) and lower values in flatter basins (e.g., Egla, Feiran). These results highlight the influence of geology, slope, and catchment size on the evolution and efficiency of drainage networks^63,64^.

**Table 4:** Estimated drainage network parameters of the studied sub-basins.

Sub-basin	Total stream number	Total stream length (km)	Drainage density (km/km²)	Stream frequency
Abu Trifa	18	20.33	0.97	0.86
B1	18	19.87	1.02	0.92
B2	22	24.93	1.11	0.98
B3	24	28.44	0.88	0.74
Egla	8	14.43	0.90	0.50
El Rummana	211	217.31	1.04	1.01
El-Akhder	350	347.04	1.05	1.05
El-Sheikh	390	352.97	1.06	1.18
Feiran	142	137.15	0.40	0.41
Feiran downstream	119	124.39	0.92	0.88
Hadahad	53	60.55	1.20	1.05
Khuzayra	38	33.28	0.85	0.97
Nisyrin	74	84.75	0.99	0.86
Solaf	298	303.39	0.97	0.95
Takos	36	39.88	1.06	0.96
Um Lasifa	36	39.88	1.06	0.96

Basin shape indices (Table 5) also revealed marked differences in geomorphological characteristics. Most basins were elongated (Re < 0.7; Ff < 0.5), such as B3, Feiran Downstream, and El-Akhder, which suggests slower runoff responses and longer times of concentration. By contrast, basins such as Takos, Nisryin, and Khuzayra displayed relatively higher values (Ff > 0.35; Re ≈ 0.7), implying more circular shapes and a faster hydrological response to rainfall events. Variations in circularity ratio (Rc) and compactness coefficient (Cc) further distinguished the basins: small basins such as Egla, Takos, and Khuzayra recorded relatively high Rc values (0.41–0.63), indicating more regular forms, while larger basins including El-Akhder, El-Sheikh, and Solaf recorded lower Rc (< 0.30), consistent with elongated and irregular geometries. These findings were confirmed by the length–width ratio (L/W), which reached its maximum in Feiran (4.40) and El-Sheikh (4.10), highlighting pronounced elongation compared to lower values (< 2) in Khuzayra and Nisryin. Such morphometric indices are widely recognized as key indicators of basin hydrological response^62,65^.

**Table 5:** Estimated shape parameters of the studied sub-basins

Sub-basin	Basin length (km)	Basin width (km)	Area (km²)	Perimeter (km)	Form factor	Elongation ratio	Circularity ratio	Compactness coefficient	Length -width ratio
Abu Trifa	7.32	3.76	20.88	21.11	0.39	0.70	0.59	1.30	1.95
B1	7.62	3.33	19.51	21.11	0.34	0.65	0.55	1.35	2.29
B2	9.02	3.42	22.51	24.26	0.28	0.59	0.48	1.44	2.64
B3	12.09	3.12	32.26	34.70	0.22	0.53	0.34	1.72	3.88
Egla	12.09	3.75	16.08	17.94	0.11	0.37	0.63	1.26	3.22
El Rummana	24.74	9.00	208.47	94.37	0.34	0.66	0.29	1.84	2.75
El-Akhder	32.04	10.77	331.90	142.93	0.32	0.64	0.20	2.21	2.97
El-Sheikh	30.29	7.38	331.90	148.81	0.36	0.68	0.19	2.30	4.10
Feiran	29.15	6.63	342.76	117.08	0.40	0.72	0.31	1.78	4.40
Feiran downstream	30.43	8.22	134.99	123.05	0.15	0.43	0.11	2.99	3.70
Hadahad	14.72	5.83	50.39	40.80	0.23	0.54	0.38	1.62	2.52
Khuzayra	9.05	4.78	39.26	29.89	0.36	0.68	0.41	1.56	1.89
Nisyrin	15.02	8.19	86.04	51.42	0.38	0.70	0.41	1.56	1.83
Solaf	33.59	10.61	314.31	121.42	0.28	0.60	0.27	1.93	3.17
Takos	9.60	3.81	38.15	32.55	0.41	0.73	0.45	1.49	2.52
Um Lasifa	11.34	3.68	37.55	33.74	0.29	0.61	0.41	1.55	3.08

Relief parameters (Table 6) provided additional insight into the topographic variability of the basin. Basins such as El-Sheikh, Solaf, and Egla exhibited very high relief values (> 1400 m), reflecting steep terrain, tectonic influence, and high erosional energy. In contrast, B3, Um Lasifa, and Feiran Downstream displayed relatively low relief (< 400–800 m), indicative of subdued topography and lower erosion intensity. The relief ratio (Rr) was highest in smaller, steeper basins such as Egla, Takos, and Abu Trifa, pointing to rapid vertical erosion, while larger and flatter basins (e.g., Feiran Downstream, Um Lasifa, El-Akhder) recorded low ratios, suggesting slower dissection. Similarly, the ruggedness number (Rn) emphasized sharp contrasts: extremely high values were observed in El-Sheikh, Solaf, and Egla (> 1200), highlighting their rough terrain and high runoff potential, while smaller basins such as Um Lasifa and B3 recorded very low values (< 350), reflecting relatively stable geomorphic surfaces^61,66^.

**Table 6:** Estimated relief parameters of the studied sub-basins.

Sub-basin	Min elev. (m)	Max elev. (m)	Relief (m)	Relief ratio	Ruggedness number
Abu Trifa	268.10	949.20	681.10	93.05	663.16
B1	214.00	695.40	481.40	63.18	490.28
B2	377.10	958.90	581.80	64.50	644.35
B3	104.00	491.90	387.90	32.08	341.97
Egla	645.40	2063.80	1418.00	117.29	1272.50
El Rummana	397.60	1484.50	1086.90	43.93	1132.99
El-Akhder	740.10	1620.20	880.10	27.47	920.45
El-Sheikh	766.40	2617.50	1851.10	61.11	1968.61
Feiran	309.50	1972.30	1662.80	57.04	665.34
Feiran downstream	4.60	770.60	766.00	25.17	705.85
Hadahad	127.80	651.40	523.60	35.57	629.17
Khuzayra	193.60	743.40	549.80	60.72	672.25
Nisyrin	344.90	1209.90	865.00	57.59	852.03
Solaf	759.50	2616.70	1857.20	55.27	1784.05
Takos	584.10	1533.20	929.10	96.78	828.76
Um Lasifa	149.90	452.50	302.60	26.68	321.38

Collectively, the morphometric analysis demonstrates that basins with high relief and ruggedness (e.g., El-Sheikh, Solaf) are most vulnerable to flash floods and slope instability due to their high terrain energy, steep gradients, and rough surfaces. In contrast, smaller, less dissected basins such as Um Lasifa and B3 exhibit more subdued hydrological responses. These findings reinforce the strong interdependence between geomorphological setting, basin morphology, and hydrological dynamics, and they provide essential context for interpreting groundwater recharge potential, runoff behavior, and pollution susceptibility in the Wadi Feiran Basin.

### Groundwater parameter variability and its implications

**Fig. 5:**
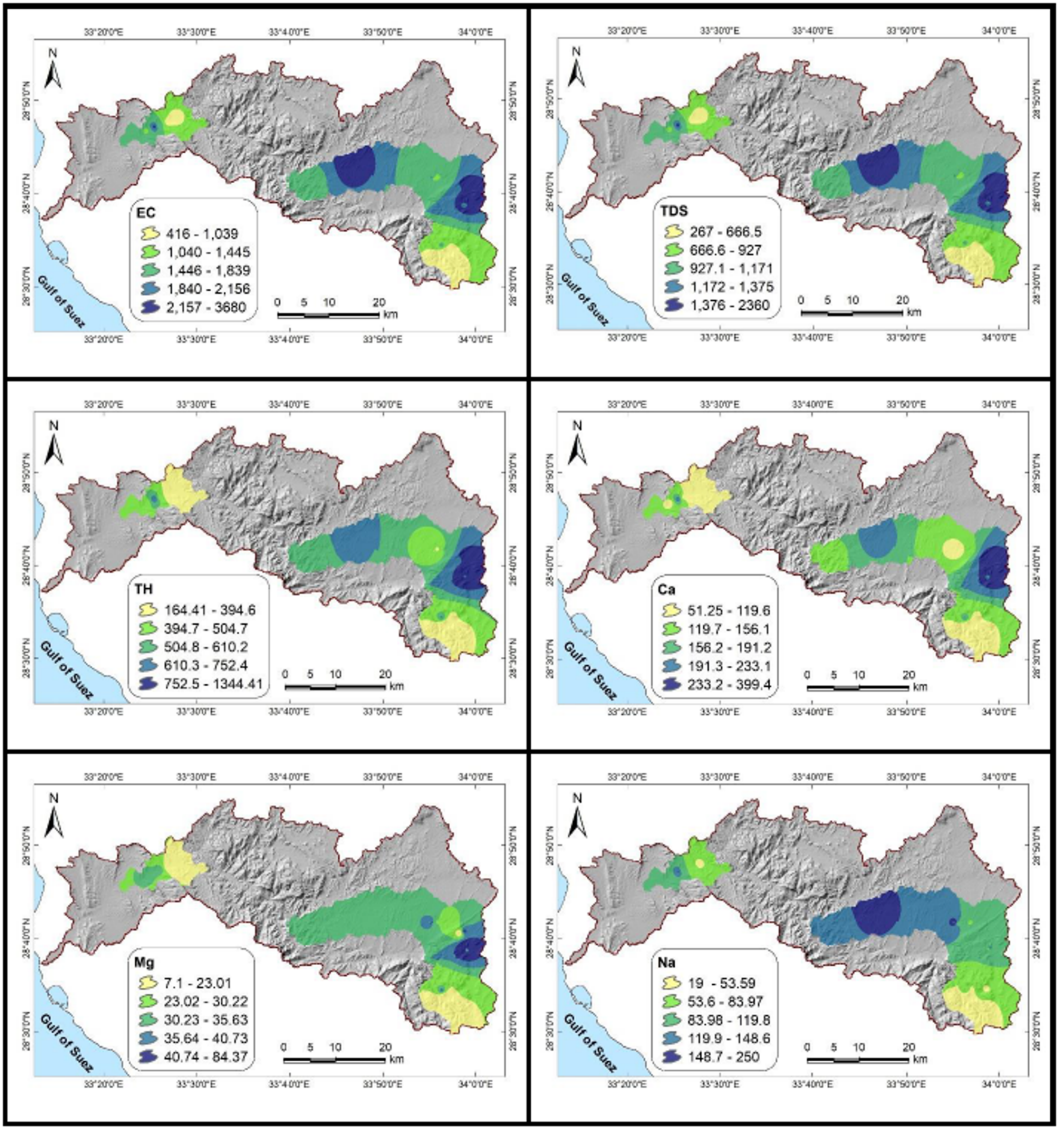

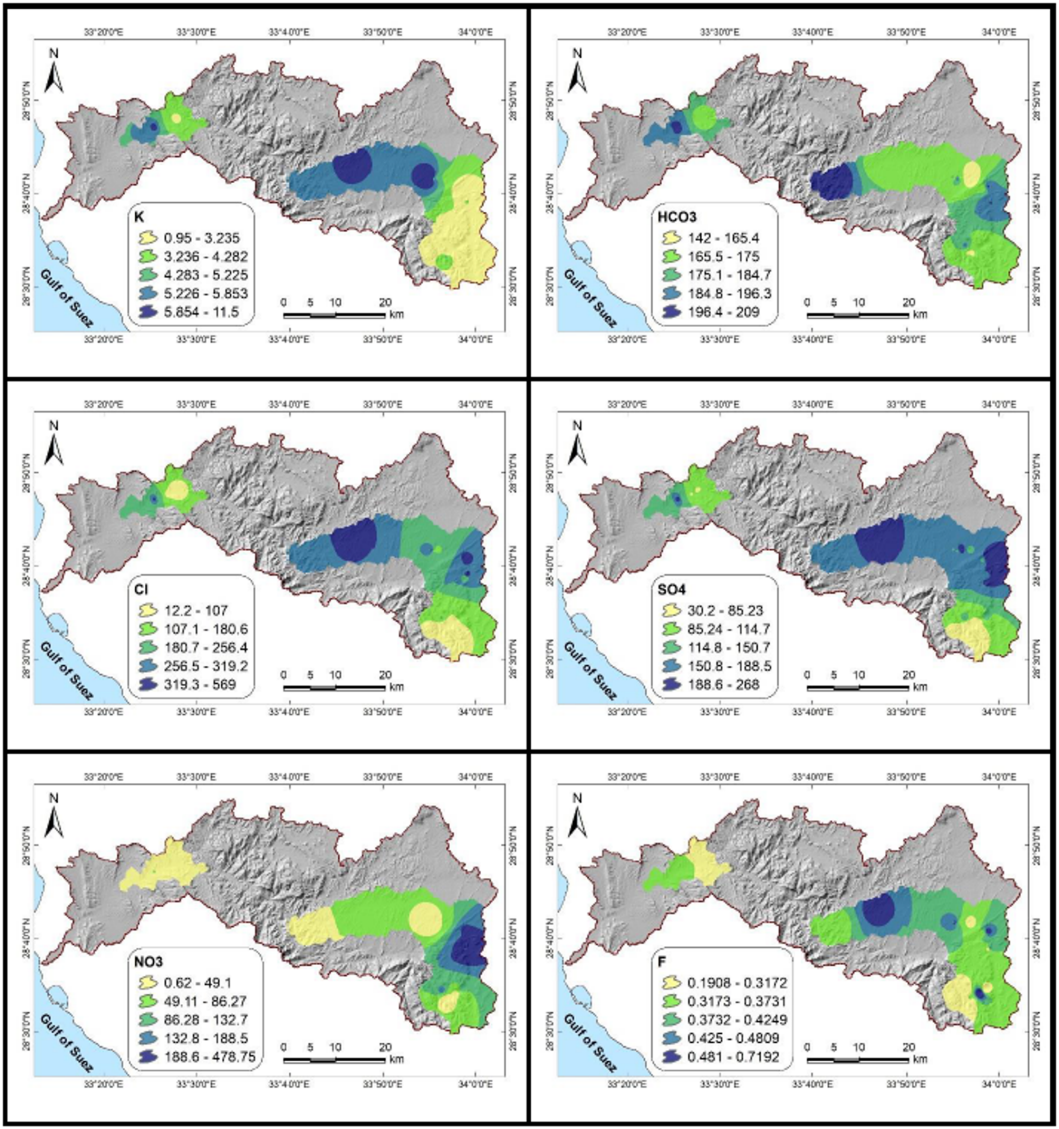

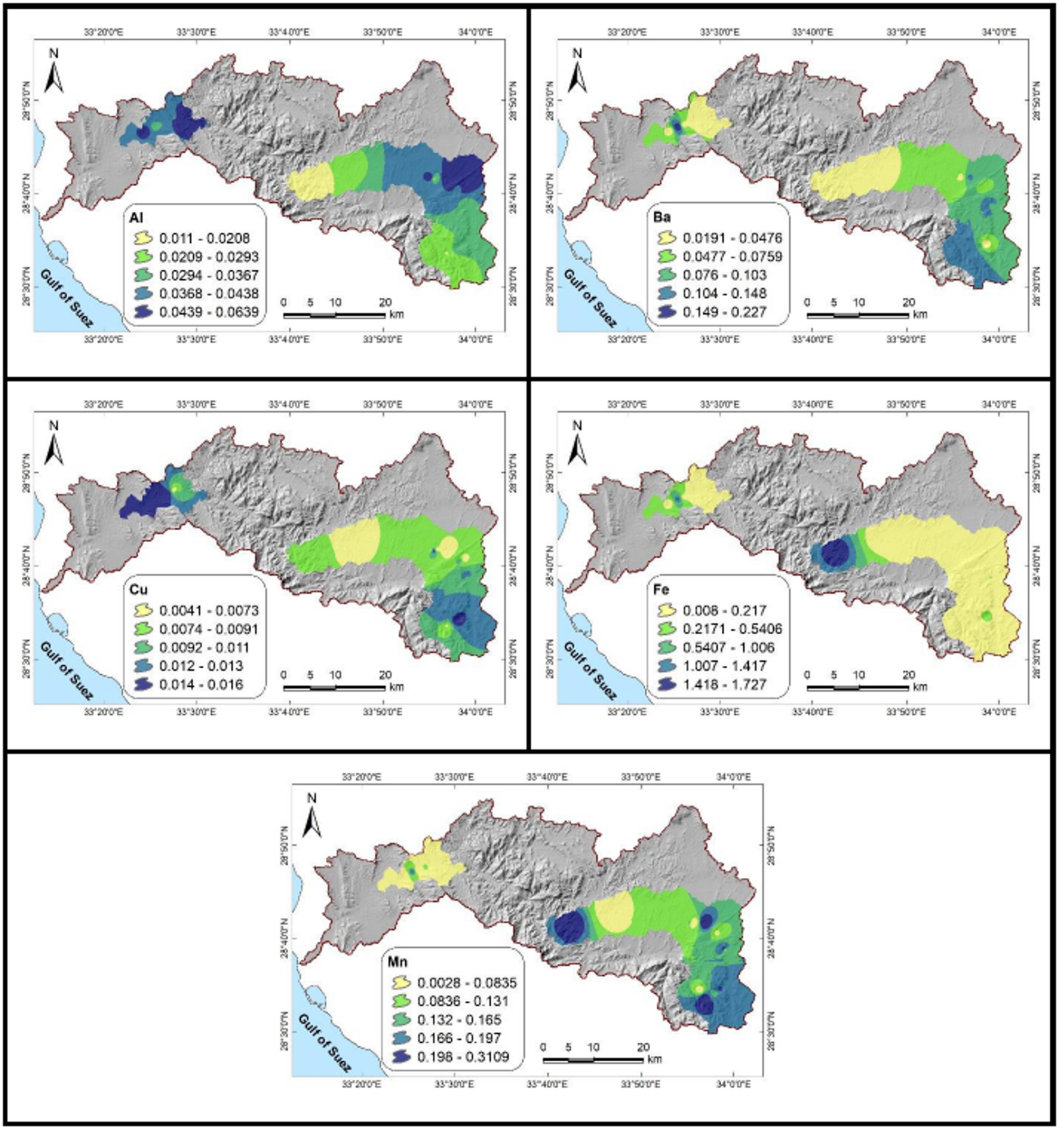
Spatial distribution maps of groundwater quality parameters in the Wadi Feiran Basin. EC is expressed in μS/cm, while all other elements and parameters are presented in ppm. All maps were created using ArcGIS Desktop v. 10.8, with a background DEM sourced from the USGS Earth Explorer portal (https://earthexplorer.usgs.gov/)

There is significant variation in groundwater quality (Fig. 5; Table 7). pH values between 7.35 and 8.20 show a slight alkalinity from carbonate buffering, favoring irrigation and household use while limiting the mobility of heavy metals^14,50^. With lower values (< 1000 µS/cm) indicating freshwater and higher values associated with salinity from mineral dissolution or human inputs, EC ranges from 416 to 3680 µS/cm^50,67^. Accordingly, TDS ranges from 267 to 2360 ppm, with less than 500 ppm safe for consumption and more than 1000 ppm possibly needing treatment^68^.

**Table 7:** Descriptive statistics of the data collected from the groundwater samples.

Variable		Min.	Max.	Average	Range	WHO guidelines
pH		7.4	8.2	0.9	7.8	6.5–8.5
EC (µS/cm(		416	3680	3264	1555	1500
TDS (ppm)		267	2360	2093	995.7	1000
TH (ppm)		164	1344	1180	509	500
Ca^2+^ (ppm)		51	399	348	148	75
Mg^2+^ (ppm)		7	84	77	28	100
Na^+^ (ppm)		19	250	231	92	250
K^+^ (ppm)		0.95	11.50	10.55	4.08	12
HCO_3_^−^ (ppm)		142	209	67	177	250
Cl^−^ (ppm)		12.2	569.0	556.8	197.7	250
SO_4_^2−^ (ppm)		30.2	268.0	237.8	141.4	250
NO_3_^−^ (ppm)		0.6	478.8	478.1	81.3	50
F^−^ (ppm)		0.19	0.72	0.53	0.36	1.5
Al (ppm)		0.011	0.064	0.053	0.03636	0.2
Ba (ppm)		0.01391	0.2270	0.2131	0.07702	1.3
Cu (ppm)		0.0041	0.0162	0.0121	0.01006	2.0
Fe (ppm)		0.008	1.727	1.719	0.201	0.3
Mn (ppm)		0.0028	0.3109	0.3081	0.1306	0.4
Saturation indices	Anhydrite	−3.525	−1.045	2.48	−1.718	-
	Aragonite	0.181	0.867	0.686	0.4733	-
	Calcite	0.325	1.011	0.686	0.6172	-
	Dolomite	0.352	1.684	1.332	0.8762	-
	Fluorite	−2.502	−1.272	1.23	−1.958	-
	Gypsum	−3.275	−0.795	2.48	−1.468	-
	Halite	−8.148	−5.521	2.627	−6.536	-
	Sylvite	−9.051	−6.439	2.612	−7.456	-

Most samples are classified as hard water (> 150 ppm) based on TH, which ranges from 164 to 1344 ppm and has implications for scaling. Ca^2+^ (51.25–399.40 ppm), Mg^2+^ (7.10–84.37 ppm), Na^+^ (19–250 ppm), and K^+^ (0.95–11.50 ppm) are the main cations that are produced by fertilizer input, saline intrusion, and mineral dissolution^2,69–71^. HCO₃⁻ (142–209 ppm), Cl⁻ (12.2–569 ppm), SO₄²⁻ (30.2–268 ppm), and NO₃⁻ (0.62–478.80 ppm) are examples of anions. Significant health risks were indicated by eleven samples that had nitrate levels above the WHO limit (> 50 ppm)^2,72^. Safe levels of fluoride (0.19–0.72 ppm) are maintained^72^.

**Fig. 6:**
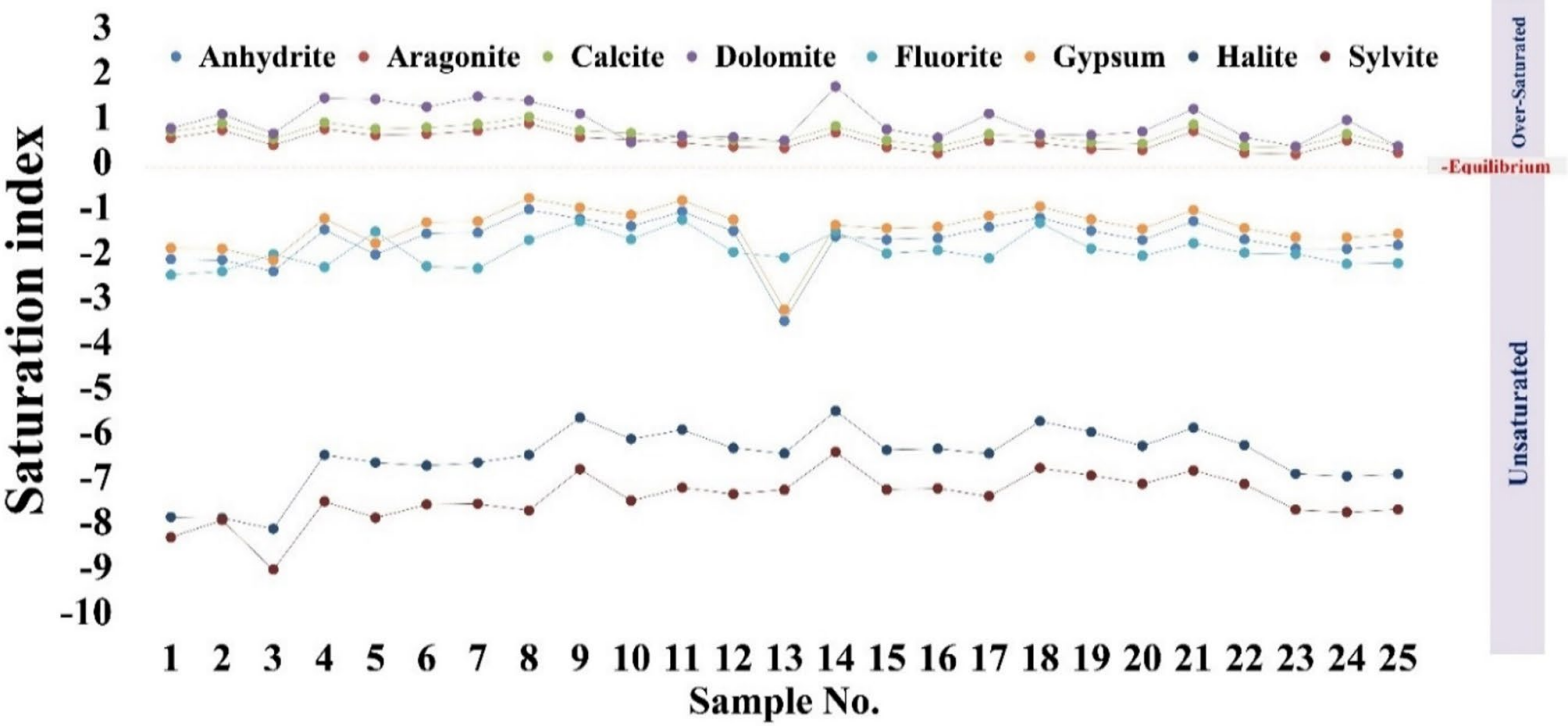
Saturation indices of dissolved minerals in the studied groundwater samples.

Though Fe (0.008–1.727 ppm) surpasses the WHO limit (0.30 ppm) in two samples, indicating localized mineral weathering, trace metal levels are generally low. The elements Al, Ba, Cu, and Mn continue to be compliant. Undersaturation of anhydrite, gypsum, halite, and sylvite indicates dissolution, while oversaturation of aragonite, calcite, and dolomite indicates active precipitation, according to saturation indices (Table 8; Fig. 6).

**Table 8:** PCA loadings for variables in groundwater samples.

Variables	PC1	PC2	PC3	PC4
pH	−0.46	0.74	−0.32	0.22
EC	0.98	0.00	−0.03	−0.16
TDS	0.98	0.00	−0.03	−0.16
TH	0.91	0.06	0.22	−0.33
Ca^2+^	0.85	0.09	0.32	−0.38
Mg^2+^	0.84	0.04	−0.11	−0.05
Na^+^	0.83	−0.10	−0.45	0.24
K^+^	0.35	−0.07	−0.69	0.43
HCO_3_^−^	0.43	−0.05	0.60	0.38
Cl^−^	0.95	−0.04	−0.23	−0.10
SO_4_^2−^	0.79	0.02	0.09	0.12
NO_3_^−^	0.64	0.22	0.40	−0.54
F^−^	0.52	−0.03	−0.44	0.04
Al	0.18	−0.33	−0.31	−0.36
Ba	0.05	0.66	−0.18	−0.34
Cu	0.09	0.01	0.14	0.33
Fe	0.27	0.14	0.06	0.34
Mn	−0.03	0.41	−0.41	−0.48
Anhydrite	0.68	0.11	0.44	0.37
Aragonite	0.19	0.94	0.06	0.05
Calcite	0.19	0.94	0.06	0.05
Dolomite	0.22	0.85	−0.16	0.28
Fluorite	0.79	−0.12	−0.18	−0.12
Gypsum	0.67	0.11	0.44	0.37
Halite	0.89	−0.23	−0.18	0.16
Sylvite	0.79	−0.24	−0.32	0.26
Eigenvalue	10.77	4.02	2.63	2.24
Variance (%)	41.42%	15.48%	10.10%	8.61%
Cumulative (%)	41.42%	56.90%	67.00%	75.61%

According to earlier studies^6,11,73^, overall exceedances in EC, TDS, TH, Ca^2+^, Cl⁻, SO₄²⁻, NO₃⁻, and Fe indicate possible hazards for irrigation and drinking water. These results highlight the necessity of ongoing observation and groundwater management tailored to a given area.

### Geochemical mechanisms and groundwater facies distribution

**Fig. 7:**
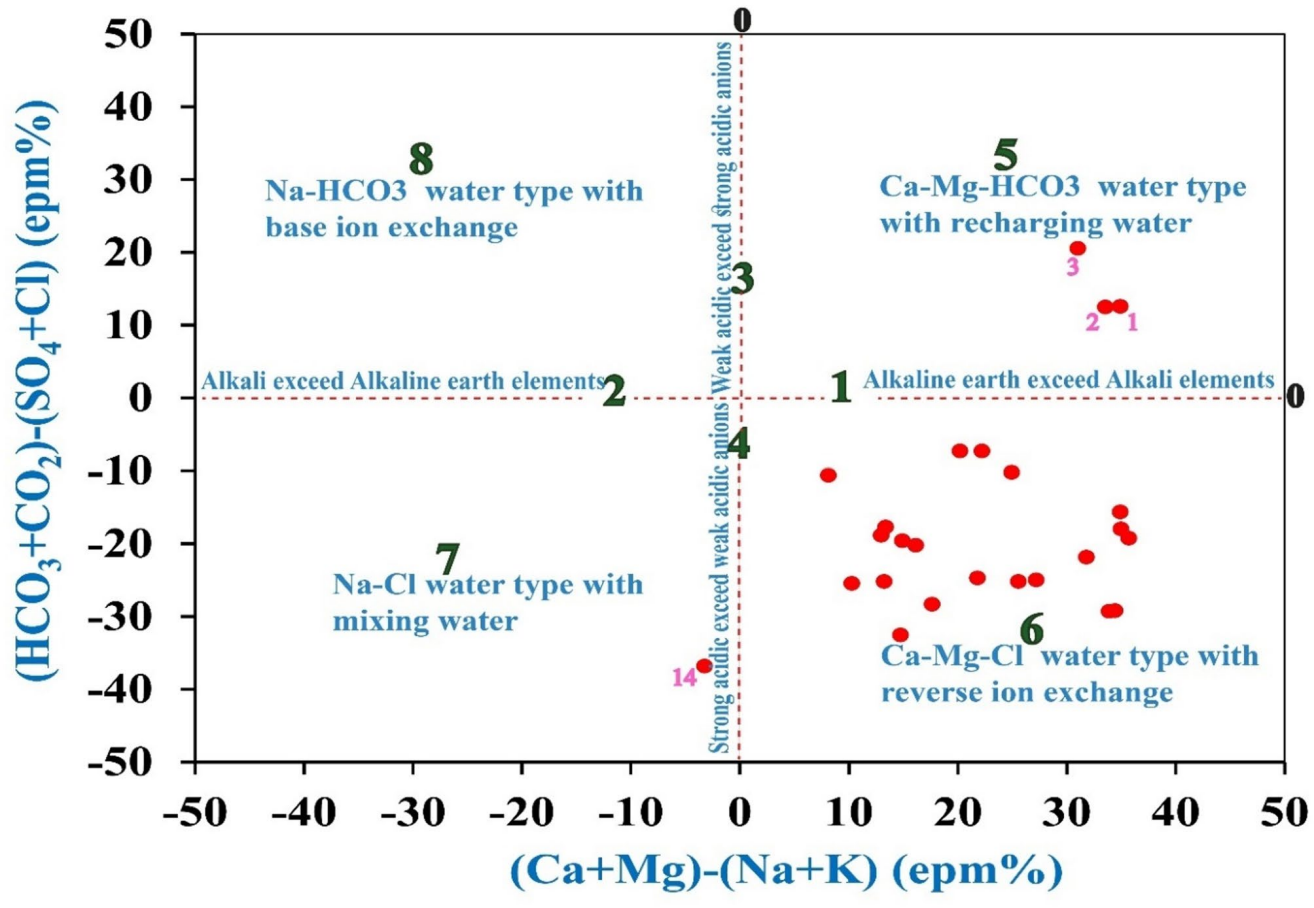
The Chadha diagram illustrates the hydrochemical characteristics of the studied groundwater.

According to the Chadha diagram (Fig. 7)^74^, one sample plots in field 2, which is dominated by alkali, while 24 samples fall in field 1, which is dominated by alkaline earths (Ca^2+^ and Mg^2+^). Three samples in field 3 exhibit the opposite trend, while twenty-two samples in field 4 exhibit strong acid prevalence (Cl⁻, SO₄²⁻). These trends demonstrate how ion exchange and evaporite dissolution play a part in groundwater chemistry^50,75^. Three Ca-Mg-HCO₃ samples (field 5) show freshwater signatures, and 21 Ca-Mg-Cl samples (field 6) validate freshening and cation exchange. One type of Na-Cl (field 7) indicates anthropogenic salinization or seawater intrusion^76^. Precipitation recharge and strong geochemical evolution are confirmed by such variability, which is consistent with previous studies^11,73^.

**Fig. 8:**
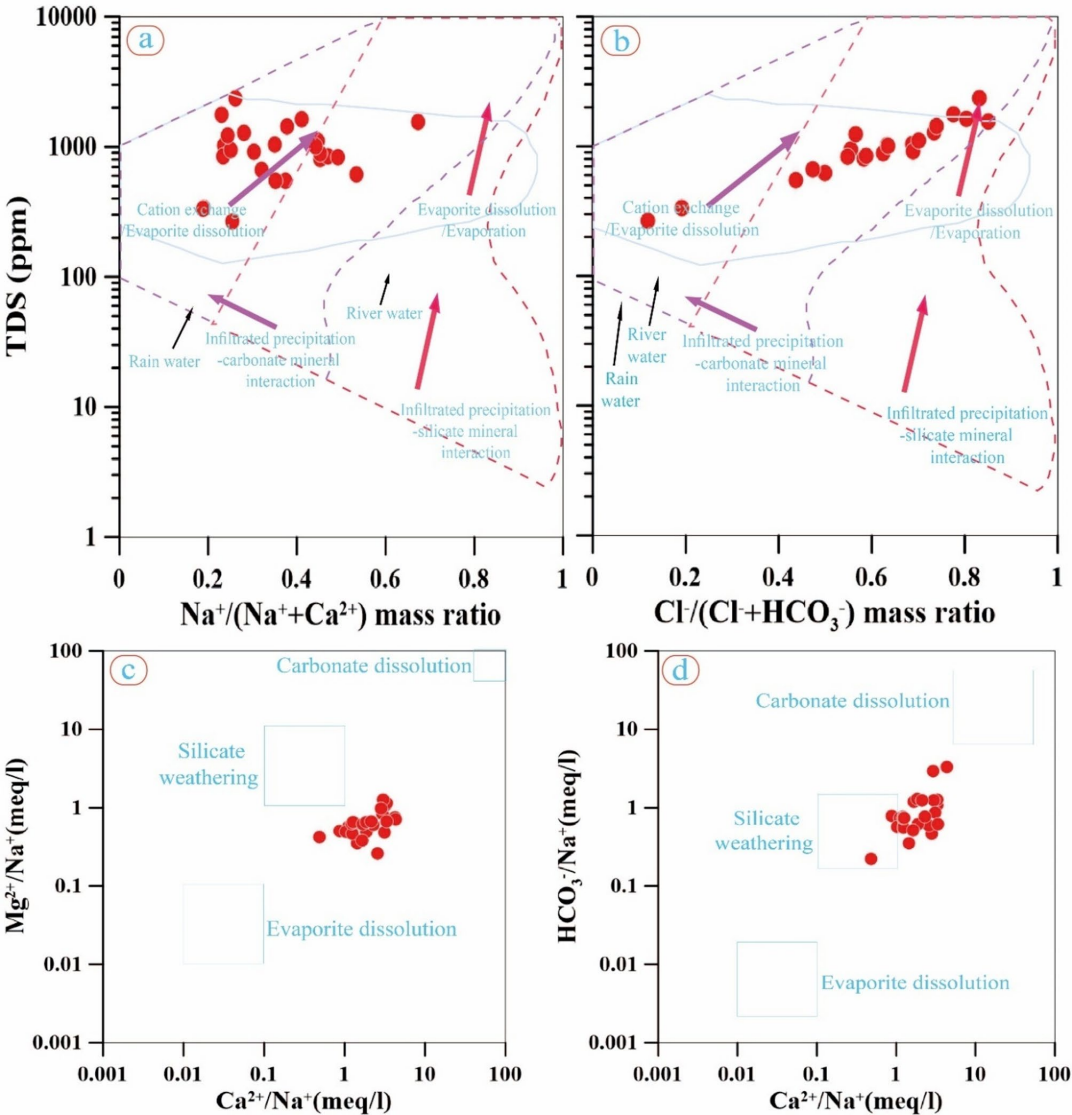
(**a**, **b**) The Gibbs plots and (**c**, **d**) the End-member graphs provide a detailed representation of the hydrochemical processes occurring in the studied groundwater, highlighting the influences of different water-rock interactions and environmental factors on water chemistry.

The Gibbs and End-member plots (Fig. 8) show that evaporite dissolution and cation exchange are secondary processes to silicate weathering^50,77–79^. There is also a slight dissolution of carbonate. These results are consistent with processes observed in other groundwater aquifers in Sinai^6,11,73^, and directly support the study’s objective of linking groundwater quality to its controlling mechanisms.

In conclusion, Figs. 7, 8 and Tables 7, 8 show that both natural processes and outside influences influence the chemistry of groundwater. Ion exchange, evaporite dissolution, and sporadic seawater intrusion are processes that are regionally dominant when compared to recent Sinai studies. These revelations highlight the necessity of ongoing management and monitoring to safeguard groundwater against contamination and salinity threats.

### Impact of geochemical processes on groundwater quality

**Fig. 9:**
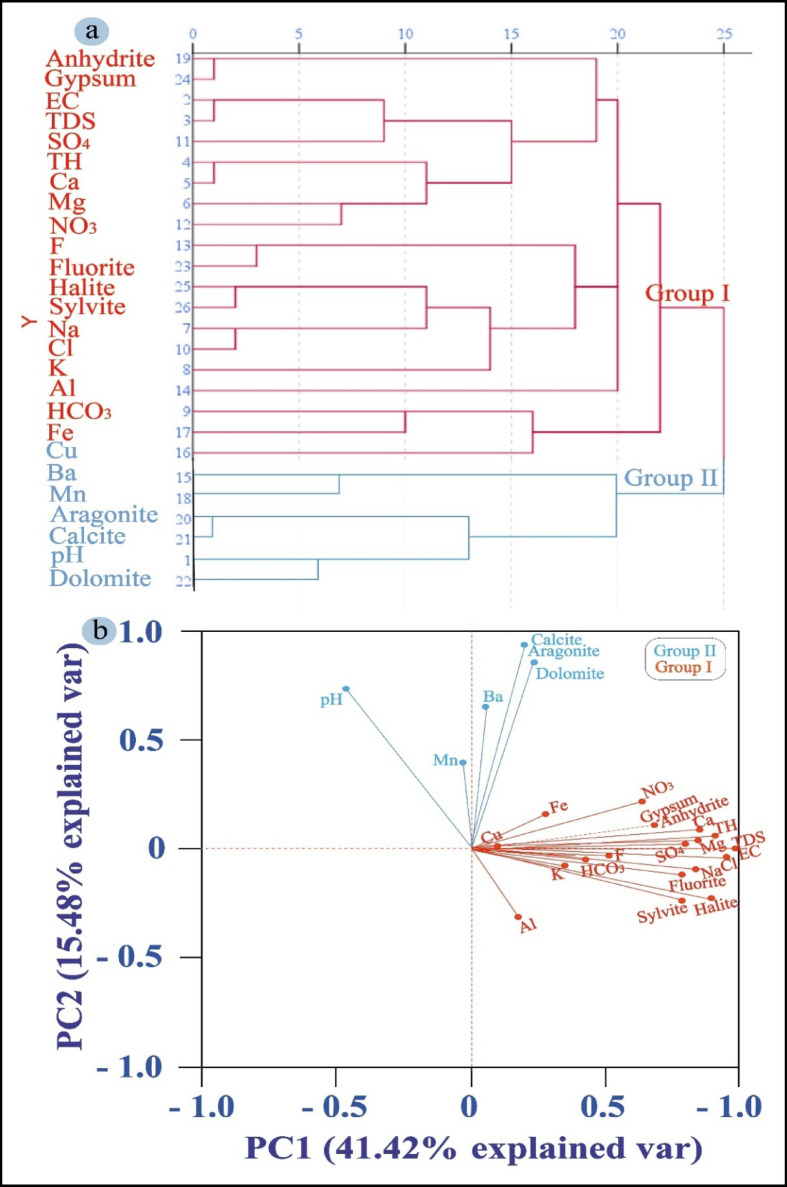
(**a**) The dendrogram graph and (**b**) the PCA analysis offer insights into the hydrochemical processes occurring in the studied groundwater, helping to identify and classify the key factors influencing water composition and quality.

Groundwater parameters are grouped into two clusters by the dendrogram (Fig. 9a). Group II shows limited carbonate solubility with influences from Ba, Mn, and Cu, whereas Group I shows mineralization from evaporite dissolution and fertilizer-derived NO₃⁻. This distinction demonstrates how groundwater chemistry is shaped by both natural and man-made processes, with nitrate contamination emerging as a major concern.

These controls are further clarified by PCA results (Table 8; Fig. 9b). The dendrogram results are confirmed by PC1 (41.42% variance), which records carbonate and evaporite dissolution with ion exchange. The trace elements (Ba, Mn, and Cu) with a minor carbonate role are highlighted in PC2 (15.48%). PC4 (8.61%) indicates localized trace metal enrichment (Cu, Fe) and bicarbonate buffering with reduced nitrate influence, while PC3 (10.10%) reflects anthropogenic inputs from fertilizers and potash in combination with evaporite–carbonate interactions. Together, these components demonstrate that groundwater quality is shaped by both geological processes and human activities.

The analysis directly supports the study’s objective of identifying the controls on groundwater chemistry by distinguishing natural processes from agricultural and contamination impacts through the integration of clustering and PCA. Comparable results have been reported in recent studies from Sinai^6,11,73^, which emphasize the dominance of nitrate inputs, ion exchange, and evaporite dissolution. These parallels highlight the regional significance of fertilizer-driven nitrate enrichment and salinity. Overall, these findings not only place the results within a broader regional context but also underscore the urgent need for targeted groundwater management strategies.

### Groundwater quality evaluation via NPI

With the highest loads from NO₃⁻ (0.01–9.6) and Fe (0.03–5.76), followed by Ca²⁺ and Cl⁻, Table 9 displays a wide range of Pollution Index (Pi) values. According to the ranking (NO₃⁻ > Fe > Ca²⁺ > Cl⁻ > SO₄²⁻ > Na⁺ > K⁺ > Mg²⁺ > HCO₃⁻ > Mn > F⁻ > Al > Ba > Cu), nitrate and iron are the most common pollutants, primarily associated with fertilizer use, wastewater, and industrial inputs rather than natural processes^44–46^.

**Table 9:** Pollution Index (Pi) values for variables in the groundwater samples

Variable	Min.	Max.	Range	Mean
pH	0.86	0.96	0.10	0.91
EC	0.28	2.45	2.18	1.04
TDS	0.27	2.36	2.09	1.00
TH	0.33	2.69	2.36	1.02
Ca^2+^	0.68	5.33	4.64	1.97
Mg^2+^	0.07	0.84	0.77	0.29
Na^+^	0.08	1.00	0.92	0.37
K^+^	0.08	0.96	0.88	0.34
HCO_3_^−^	0.57	0.83	0.27	0.71
Cl^−^	0.05	2.28	2.23	0.79
SO_4_^2−^	0.12	1.07	0.95	0.57
NO_3_^−^	0.01	9.58	9.56	1.63
F ^−^	0.13	0.48	0.35	0.24
Al	0.06	0.32	0.27	0.18
Ba	0.01	0.17	0.16	0.06
Cu	0.00	0.01	0.01	0.01
Fe	0.03	5.76	5.73	0.67
Mn	0.01	0.78	0.77	0.33

60% of samples are unsafe for consumption, according to NPI values (Table 9), with 4% being severely polluted, 12% being moderately polluted, 20% being lightly polluted, and 24% being slightly polluted. These findings are consistent with recent studies from the Sinai Peninsula^6,11,46,73^, which likewise identify salinity and nitrate as persistent threats to groundwater quality.

All things considered, the information directly relates to the study’s goal of assessing groundwater vulnerability. To protect public health and ensure sustainable groundwater use, fertilizer regulation, wastewater treatment, and routine monitoring are critically needed, as highlighted by the prevalence of anthropogenic pollution.

### Human health risk assessment for nitrate contamination

**Fig. 10:**
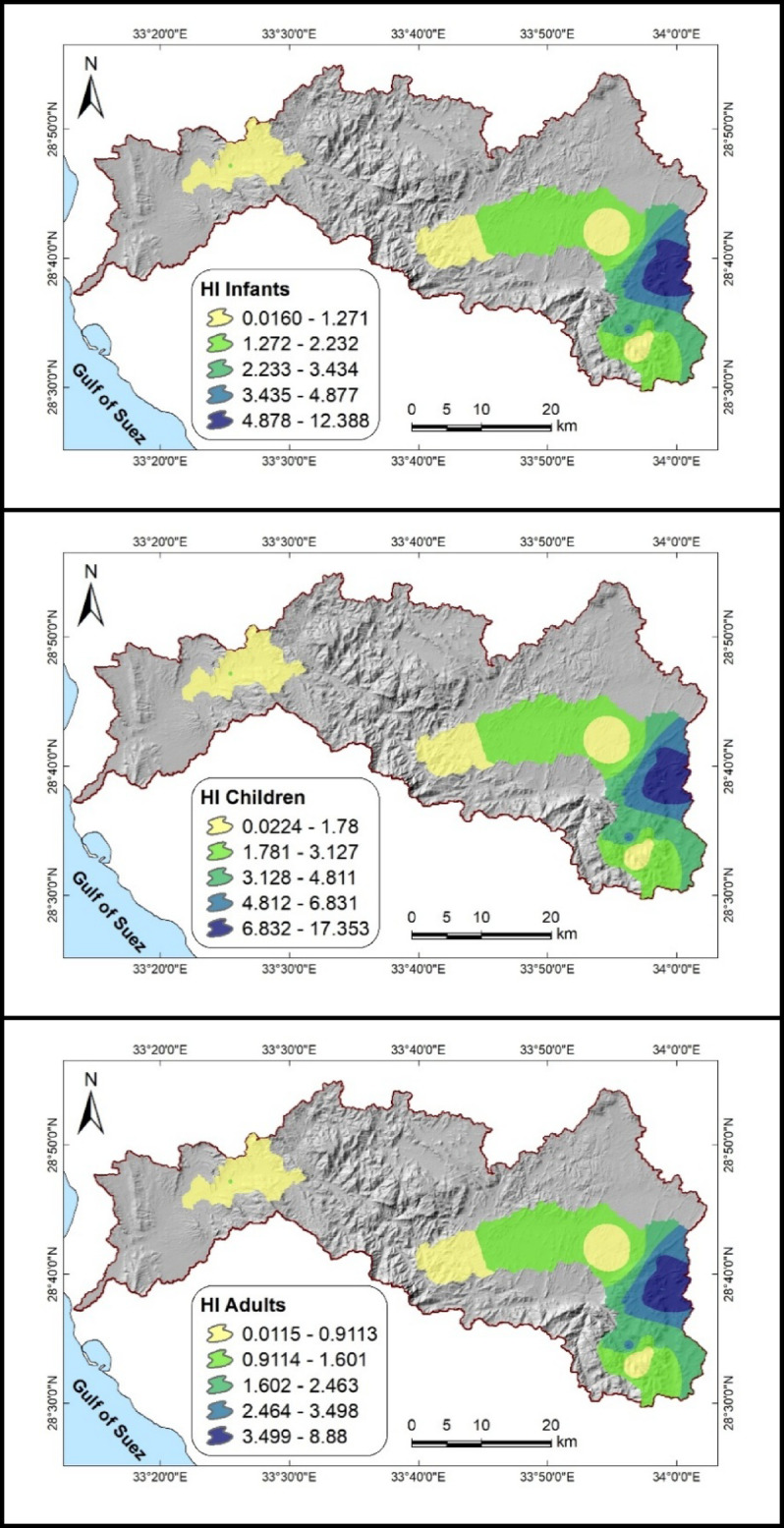
Distribution maps of HI values illustrating non-carcinogenic risk levels for infants, children, and adults in the Wadi Feiran Basin. All maps were created using ArcGIS desktop v. 10.8, with a background DEM sourced from the USGS earth explorer portal (https://earthexplorer.usgs.gov/).

Groundwater nitrate exposure poses serious health risks, with infants and children emerging as the most vulnerable groups. As shown in Table 10 and Fig. 10, Hazard Quotient (HQ) and Hazard Index (HI) values for these groups far exceed the safe limit of 1.0, reaching up to 17.22 for children and 12.39 for infants, compared to 8.88 for adults. Oral ingestion is the dominant exposure route, while dermal pathways remain negligible. These findings align with previous studies in Sinai^6,11,73^, and similar international evidence^80,81^.

**Table 10:** The HHRA values in the groundwater samples studied.

		Min.	Max.	Range	Mean
Oral pathway	CDI_Adults_	0.01829	14.13	14.11	2.397
	CDI_Children_	0.03567	27.54	27.51	4.674
	CDI_Infants_	0.02548	19.67	19.65	3.339
	HQ_Adults_	0.01143	8.828	8.817	1.498
	HQ_Children_	0.0223	17.22	17.19	2.922
	HQ_Infants_	0.01593	12.3	12.28	2.087
Dermal pathway	CDI_Adults_	5.85E-05	0.0452	0.04514	0.007671
	CDI_Children_	0.000143	0.1102	0.11	0.0187
	CDI_Infants_	9.51E-05	0.07345	0.07336	0.01247
	HQ_Adults_	7.32E-05	0.0565	0.05643	0.009588
	HQ_Children_	0.000178	0.1377	0.1375	0.02337
	HQ_Infants_	0.000119	0.09182	0.0917	0.01558
Hazard index	HI_Adults_	0.01151	8.885	8.873	1.508
	HI_Children_	0.02247	17.35	17.33	2.945
	HI_Infants_	0.01604	12.39	12.37	2.102

Beyond the numerical outcomes, the results reveal critical policy and management implications. High nitrate risks highlight gaps in regulatory enforcement and the urgent need for age-sensitive monitoring, stricter water quality standards, and provision of safe alternatives for drinking water. Where nitrate already exceeds permissible limits, treatment technologies such as ion exchange and reverse osmosis should be considered.

Agricultural runoff remains the primary source of nitrate pollution, underlining the importance of better nutrient management practices. Strategies such as reducing fertilizer application, adopting precision farming, and implementing vegetative buffer strips could mitigate contamination while supporting sustainable agriculture.

At the community level, awareness campaigns are essential to communicate the dangers of nitrate exposure, especially risks such as methemoglobinemia (“blue baby syndrome”). Finally, nitrate must be treated not only as an acute hazard but also as a long-term carcinogenic risk due to its conversion into nitrosamines, which are associated with gastrointestinal cancers^82,83^.

### Identification of hotspot areas

The results of the AHP analysis are summarized in Tables 11, 12. Among the four criteria, nitrate concentration (NO₃⁻) received the highest weight (36.3%), underscoring its central role as the dominant geochemical driver of groundwater-related health risks. This finding is consistent with numerous studies identifying nitrate as one of the most pervasive groundwater contaminants in arid and semi-arid regions^84^. Population density (32.6%) ranked second, reflecting its direct link to exposure and vulnerability, in line with studies that highlight demographic pressure as a key determinant of groundwater contamination risks^55^. Distance from watercourses (16.3%) was ranked third, consistent with its role in recharge and contaminant transport pathways, while distance from urban areas (14.8%) had the lowest weight, indicating more localized anthropogenic impacts.

**Table 11:** Relative weights of groundwater health risk factors derived from the analytic hierarchy process (AHP) in the Wadi Feiran Basin.

Rank	Factor	Weight (%)	Sensitivity (±%)
1	Nitrate concentration	36.3	5.5
2	Population density	32.6	2.7
3	Distance from streams	16.3	1.4
4	Distance from urban areas	14.8	2.0

**Table 12:** Pairwise comparison matrix of groundwater contamination risk factors using the AHP.

Layer	Nitrate (NO₃⁻)	Population density	Distance from streams	Distance from urban areas
Nitrate (NO₃⁻)	1.00	1.00	2.00	3.00
Population density	1.00	1.00	2.00	2.00
Distance from streams	0.50	0.50	1.00	1.00
Distance from urban areas	0.33	0.50	1.00	1.00

The pairwise comparison matrix supported this hierarchy: nitrate and population density were consistently rated as more important than the other factors, while watercourses and urban proximity were assigned relatively lower importance. Sensitivity analysis revealed only minor changes in weights (± 1.4–5.5%), confirming the robustness of the model and reliability of the calculated weights.

**Fig. 11:**
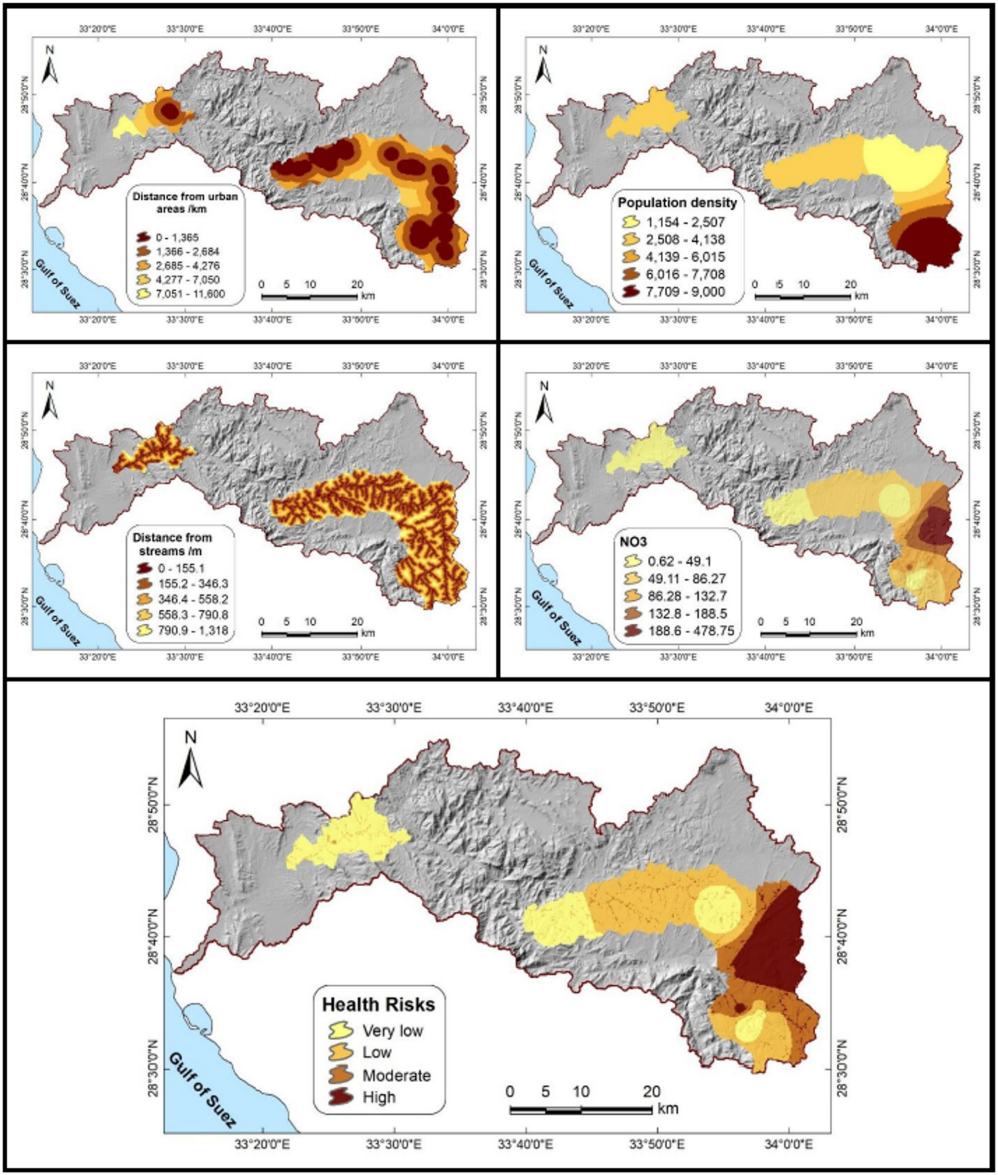
The upper panels show the distance-from-urban-areas layer (left) and the population density layer (right). The middle panels present the distance-from-streams layer (left) and the nitrate concentration (right). The lower panel shows the composite groundwater health risk map, generated through the AHP by integrating these four layers across the study area.

The composite risk map (Fig. 11) produced from the weighted overlay of these factors highlights the spatial variability of groundwater health risks across the basin. Very low-risk areas are mainly located in the western and northwestern parts of the basin, remote from dense settlements and intensive human activities. Low to moderate risk zones are concentrated in the central basin, where anthropogenic pressures are limited. In contrast, the highest risk levels are concentrated in the eastern and southeastern sectors, particularly around Saint Catherine, where high nitrate concentrations overlap with dense population, expanding urban activity, and proximity to streams. These overlapping conditions create distinct hotspots that should be prioritized in groundwater management, pollution control, and health protection strategies.

These results reinforce the critical role of nitrate pollution and demographic pressures in shaping groundwater health risks, consistent with recent findings from the Sinai Peninsula^6,11,73^, and comparable arid environments in South Asia^80,81^. The integrated AHP-based approach demonstrates that combining geochemical and socio-environmental layers can provide valuable insights for sustainable groundwater management and policy development in vulnerable arid regions. Overall, these findings underscore the need for an integrated framework that combines preventive agricultural practices, targeted treatment technologies, and public health policies to ensure safe and sustainable groundwater use in arid regions such as Wadi Feiran.

## Conclusions

This study integrated morphometric, hydrochemical, multivariate statistical, and health risk assessments to evaluate groundwater quality and watershed dynamics in the Wadi Feiran Basin, Southwestern Sinai. The findings reveal that the basin’s steep relief and high drainage density accelerate surface runoff, creating both flood risks and opportunities for water harvesting.

Groundwater quality shows substantial variability, with EC, TDS, TH, and nitrate concentrations frequently exceeding WHO limits. Hydrochemical facies indicate that rock–water interactions, silicate weathering, and evaporite dissolution dominate water chemistry, while agricultural runoff and wastewater infiltration intensify nitrate pollution. Pollution indices classify 60% of samples as unsafe for human consumption.

Health risk analysis highlights nitrate as the critical contaminant, with children facing the greatest non-carcinogenic risks. Elevated Hazard Quotient and Hazard Index values underscore the urgency of intervention.

These results have clear implications for water resource management. Effective measures should include stricter control of agricultural runoff, improved wastewater treatment, and advanced nitrate/iron removal techniques. Policy actions must prioritize sustainable land-use planning, enforcement of environmental regulations, and community awareness campaigns.

Future research should focus on long-term monitoring of groundwater quality, climate change impacts on recharge processes, and the development of integrated water harvesting and treatment solutions tailored for arid environments.

## Data Availability

The datasets used and/or analysed during the current study available from the corresponding author “Rashad Sawires” at [rashad.sawires@aun.edu.eg], on reasonable request.
